# Reflection of Challenges and Opportunities within the COVID-19 Pandemic to Include Biological Hazards into DRR Planning

**DOI:** 10.3390/ijerph18041614

**Published:** 2021-02-08

**Authors:** Emily Ying Yang Chan, Caroline Dubois, Ada Ho Yin Fong, Rajib Shaw, Ranit Chatterjee, Ambika Dabral, Antonia Loyzaga, Yong-kyun Kim, Kevin Kei Ching Hung, Chi Shing Wong

**Affiliations:** 1 Collaborating Centre for Oxford University and CUHK for Disaster and Medical Humanitarian Response (CCOUC), The Chinese University of Hong Kong, Hong Kong, China; kevin.hung@cuhk.edu.hk (K.K.C.H.); cswong@cuhk.edu.hk (C.S.W.); 2 Nuffield Department of Medicine, University of Oxford, Oxford OX37BN, UK; 3 JC School of Public Health and Primary Care, Faculty of Medicine, The Chinese University of Hong Kong, Hong Kong, China; caroline.dubois@gxfoundation.hk; 4 GX Foundation, Hong Kong, China; fong.adahy@gmail.com; 5 Accident & Emergency Medicine Academic Unit, Prince of Wales Hospital, The Chinese University of Hong Kong, Hong Kong, China; 6 Graduate School of Media and Governance, Keio University, Fujisawa 252-0882, Japan; shaw@sfc.keio.ac.jp; 7 Resilience Innovation Knowledge Academy (RIKA), New Delhi 110059, India or chatterjee.ranit.8a@kyoto-u.ac.jp (R.C.); ambika@rikaindia.com (A.D.); 8 Graduate School of Informatics, Kyoto University, Kyoto 606-8501, Japan; 9 National Resilience Council, Pasay City 1300, Philippines; aloyzaga@observatory.ph; 10 Ministry of Interior and Safety, Sejong 30128, Korea; unkim68@gmail.com

**Keywords:** health-EDRM, disaster risk reduction, biological hazards, Sendai Framework, COVID-19 pandemic

## Abstract

COVID-19 has reinforced the need to revisit the integration of health within disaster risk reduction (DRR) strategies for biological hazards in a system-wide approach. In November 2020, DRR experts attended the Asia-Pacific Partnership for Disaster Risk Reduction (APP-DRR) Forum to share progress and learnings in the areas of health system resilience, data management, residual risk management, risk communication, digital literacy, and knowledge product marketing. Advancements for health in DRR included the importance of multi-sectoral, multi-hazard action plans; adaptation to technological advancements in data collection, dissemination and protection; promoting the health and wellbeing of essential and nonprofessional workers; and improving inclusivity in digital literacy. COVID-19 has affected progress towards the Sustainable Development Goals (SDG) and created a unique opportunity within DRR to re-evaluate the adequacy of response mechanisms against concurrent, cascading or interacting risks of future biological hazards. Health emergency disaster risk management (Health-EDRM) is a new World Health Organization paradigm that includes DRR at intra-, inter- and multidisciplinary levels. Scientific advancement under Health-EDRM is necessary for health and non-health actors in DRR education and research. Continuous education on the multifaceted risk governance is a key to building awareness, capacity and accelerating towards achieving the international DRR and the SDG targets.

## 1. Introduction

The intersection between health, resilience capacity building and disaster risk reduction (DRR) planning and strategies has emerged as an interdisciplinary field of great importance for the protection of human health and wellbeing [1] since the publication of several international frameworks, including the Sendai Framework for disaster risk reduction 2015–2030 [2], and more recently in World Health Organization (WHO) Framework for Health-Emergency Disaster Risk Management Framework (Health-EDRM) [3]. The ongoing COVID-19 pandemic has amplified the need to bring the health sector front-and-center in disaster risk management at national and international levels. A hazard is defined by the United Nations Office for Disaster Risk Reduction (UNDRR) within the Hyogo Framework for Action as “a potentially damaging physical event, phenomenon or human activity that may cause the loss of life or injury, property damage, social and economic disruption or environmental degradation. Hazards can include latent conditions that may represent future threats and can have different origins: natural (geological, hydrometeorological and biological) or induced by human processes (environmental degradation and technological hazards)” [4]. A pandemic is an example of a biological hazard, which are hazards that may be either “of organic origin or conveyed by biological vectors”, would be further defined by characteristics such as “infectiousness or toxicity, dose–response, incubation period, case fatality rate and estimation of the pathogen for transmission” [5], and may have amplified impacts in the age of globalization.

Globally, hazard management planning and response strategies have yet to reflect the non-linear transition of biological hazards, particularly pandemics, which can emerge in overlapping waves with different impacts, and the community must enter a response phase before the initial recovery phase is completed [6]. As a result, the non-linear attributes of biological hazards have rendered the human community vulnerable to protracted crises that persist and increase the community’s vulnerability to the cascading risks of multi-hazard that generate complex secondary events and interactions [7]. Many global at-risk communities face cumulative impacts of concurrent geological and hydrometeorological hazard events like earthquakes and cyclones during the COVID-19 pandemic, exacerbating existing issues of food insecurity and social security. Examples of multi-hazards with cascading risks include the cyclone Amphan landfall in India and Bangladesh, which disrupted clean water and sanitation systems, leading to barriers in adequate handwashing and hygiene, which has, in turn, exacerbated not only the spread of COVID-19 but other waterborne diseases [8]. The Philippines experiences an average of twenty tropical typhoons annually, which has added burden to the emergency situation of the country, leaving many communities to rely on their own resources for protection as local government resources are spent responding to COVID-19 [9]. In addition, many vulnerable communities are affected by syndemics—the concurrent, cascading or interacting risks of biological hazards within the same individuals and groups, thereby aggravating disease burdens such as COVID-19 and an array of noncommunicable diseases [10]. The inadequacy of available risk strategies catered for the nuances of biological hazards will undoubtedly challenge the resilience of community health and health systems.

## 2. Materials and Methods

This paper delineates the key issues as highlighted at the Asia-Pacific Partnership for Disaster Risk Reduction (APP-DRR) Forum (December 2020) convened by the United Nations Office for Disaster Risk Reduction (UNDRR) Regional office for Asia and the Pacific. This is a multi-stakeholder forum that includes governments, regional inter-governmental organizations, civil society organizations, international organizations and donor organizations. The purpose of the discussions is to monitor the implementation of the Sendai Framework across the region and for stakeholders to share community-relevant insights and identify key priority action areas towards DRR in the region [11].

The way forward for health system resilience building, data management, residual risk management, risk communications, digital literacy, and knowledge product marketing were priority areas identified in this forum and are discussed in this paper.

## 3. Results

### 3.1. Health System Resilience

When responding to a biological hazard, the health sector is expected to lead the immediate frontline response. In response to the multifaceted impacts of COVID-19, many jurisdictions mobilized resources outside of the health sector to impose control measures against the spread of the virus, which brought in travel, tourism, education, and other sectors. The most common measures included personal behavioral regulations such as mandatory face masks in public places [12]; social distancing like city lockdowns, quarantine, and school closures [13,14,15]. Other measures include emergency international travel guidelines, such as self-declaration of health status, and mandatory COVID-19 testing [16,17,18]. Although the Sendai Framework calls for the broader health system vigilance and resilience and to integrate disaster risk management across primary, secondary, and tertiary healthcare, there is a discord in how national responses are organized to respond to COVID-19. Japan, for example, has a dedicated National Action Plan for Pandemic Influenza and New Infectious Diseases (2013) [19], which provides multi-sectoral, holistic and comprehensive recommendations from the pre-outbreak to the recovery phase. The national action plan proactively monitors outbreaks in other areas of the world, and recommends international joint simulation exercises in the pre-outbreak phase. The plan contains recommendations to ensure continuity of medical care, of education, welfare-services, and business recovery mechanisms. The plan, however, does not consider concurrent, cascading or interacting hazards [6]. In contrast, Singapore’s national Pandemic Readiness and Response Plan for Influenza and Other Acute Respiratory Diseases (2014) [20] aims to mitigate the mortality and morbidity consequences after the onset of the first wave through rigid surveillance. The plan mobilizes essential services, case monitoring and isolation mechanisms, and infection control in hospitals so as to maintain healthcare provision. However, the plan is developed by the Ministry of Health and heavily emphasizes the healthcare and public health approach, but is limited in considering the role and responsibility of other sectors [6]. While these plans include guidance on post-epidemic surveillance and the lifting of social and economic restrictions to return to normalcy, neither of these plans takes into consideration the mitigation or treatment of long-term physical, social and psychological impacts of a pandemic, which can include symptoms of anxiety, depression and even posttraumatic stress among healthcare workers or the wider population [21]. In the case of COVID-19, there is evidence of post-viral syndrome, which can include fatigue, myalgia, headaches and shortness of breath [22].

Most of these communities have a primary focus on building resilient health systems and building capacity within health workers to apply DRR approaches in service delivery [2]. Nevertheless, there are broader aspects to consider:

- The capacity of the relevant health and non-health workers across the entire pathway of care should be strengthened, from screening, testing, diagnosis, treatment, recovery and rehabilitation [6];
- Surveillance and information systems must be strengthened to ensure that data collected includes all populations and will enable the system to identify and protect groups facing vulnerability [6];
- Public–private partnership models for health service provision should be explored and promoted to maximize functionality and service provision, especially when government systems are constrained [6].

### 3.2. Data Management

Biological hazards affect population groups differently, depending on their exposure. Vulnerability will vary according to the nature of the hazard, existing mitigation and protection systems, and any existing and inherent risks faced. Traditional groups facing vulnerability include, but are not limited to, older people, groups living in rural areas, migrant groups, indigenous groups, and those with comorbidities, physical or mental disabilities. In order to assess impact inclusively, baseline pre-disaster data for health and socioeconomic indicators should be made available to identify and minimize the impact of determinants that may exacerbate biological risks [6]. To achieve DRR that is inclusive of the vulnerable and the forgotten, risk assessments during emergency settings must therefore include disaggregated data and analysis for groups facing vulnerabilities such that policies may holistically address the risks of the entire community.

Meanwhile, efforts will be needed to address the challenges in data storage, monetization of data, ethics related to secondary use of data, and implications on personal protection. While sharing timely and accurate data is necessary to the response and containment to a global pandemic, stringent protocols for the secure storage and distribution of data must be implemented, which consider access rights, encryption and continuous review of security, which take into account the evolving benchmark for security and technological advancements [23]. Global data platforms such as Google, Facebook, Uber and cell phone companies have in the past monetized data, for example, in making geolocation data available to scientists for disease spread modeling or similar research. In the case of COVID-19, such information has been utilized for contact tracing or controlling population access to public spaces. In addition, a clear protocol for the management of secondary data is necessary to guarantee a balance between privacy and the usefulness of data [24]. Personal protection is important, and includes mechanisms for identity protection, protection against discrimination, understanding how personal data are used, and informed consent prior to data collection, particularly if the information collected can reveal information related to the health of the individual or their family [25].

### 3.3. Residual Risk Management

The global response to COVID-19 has highlighted essential workers as a highly exposed group with unique needs. The categorization of essential services varies between jurisdictions, across workforces from healthcare, social work, government services, agriculture, transport, waste management and others. However, despite society’s reliance on essential workers, many have been unprotected, working under inadequate health and safety conditions, and putting themselves and their families at risk [26,27]. In many communities, duties of care and protection have been undertaken by nonprofessionals such as informal home care providers [28]. There is a gendered impact of increasing reliance on informal care, which is provided by women in many communities. School closures in Asia, for example, are impacting professional women differently, who provide informal care within families. Travel restrictions have caused challenges and uncertainties to foreign domestic workers, many of whom are women [29]. Informal care providers are often not directly protected by legal measures for health protection or adequate infectious disease control training [30]. In Muslim communities, evidence has shown that women are more likely to wear face coverings in public for religious reasons, but not in their houses while caring for others, while men wear masks for hygiene both inside and outdoors [31]. In identifying and monitoring groups facing vulnerabilities, protection mechanisms can support informal care providers through alternative means like the provision of material resources such as personal protective equipment, medicines; information resources such as home care guidelines; and appropriate training so that they may be able to care for other sick or at-risk groups while minimizing their risk and exposure [6,28].

Under COVID-19, health sectors across countries have resorted to the basic means of service and functionality. The International Labor Organization has published a policy framework on protecting the workforce during the pandemic, which encompasses areas of employment stimulation, supporting enterprises, worker protection and social dialog for solutions [32]. The International Monetary Fund has conducted research on the implication of fiscal policy measures on income inequality within and between vulnerable groups, including essential workers [33]. However, there is little discussion, experience-sharing, and evidence-based lessons learned on the health impact this protract crisis has on essential or nonprofessional workers. With all the new challenges posed by megacities, migration, rural urbanization and technological advancement, what constitutes “essential” workers in a community must be revisited and defined for relevant DRR planning and capacity building.

### 3.4. Risk Communication

Risk communication can only be made effective when taking the “whole of government, whole of society” approach [3]. The global impact of biological hazards highlights the importance of effective communication between stakeholders at all levels, from the international level, among experts and policymakers, to the community level, within the general public, within households, and among individuals. As the fundamental component to enhancing community cooperation, mobilization and resilience, risk communication should include a top-down approach from government or authorities that participate in cross-country dialog to enable early and effective warning systems. These warning systems should trigger national or international standard operating procedures to mitigate the impact as early as possible [3,6]. Communication also requires the bottom-up input of the whole society to ensure that the information disseminated is tailored and relevant to all members of society and their protection. Efforts should be taken to extend this dialog to groups facing vulnerability, such as indigenous communities, migrants and refugees, for whom information transfer tends to be complex and indirect. The participation and engagement of local government, faith-based groups and religious leaders, as well as civil society groups, are essential in this process [6]. Moreover, it should be recognized that resource information channels vary with user demographics, acceptability, and access. Studies have shown that health literacy and risk perception are negatively correlated with income, education and social status. The European Health Literacy Survey conducted in eight countries demonstrated that 50% of adults have problematic or inadequate levels of access, understanding, appraisal and application of health or risk information [34,35]. A study in Australia showed that people with low health literacy and people whose native language is not English demonstrated poorer understanding of COVID-19 symptoms and prevention measures, more difficulty accessing government information, difficulty accessing prescription medication, and experienced greater anxiousness and financial difficulties [36]. Studies conducted in Australia and the United States showed that factors increasing vulnerability to COVID-19, such as age, underlying chronic diseases, and income are also factors associated with the ability to access and understand health information and decision-making [36,37]. During the COVID-19 pandemic and widespread lockdown, digital media has become a convenient and rapid tool for people to gain information. It is important that risk communication ensure equitable access and understanding by all groups and mitigate against misinformation.

### 3.5. Digital Literacy

There is growing discussion on the use and functionality of digital tools for information-sharing, contact tracing, and communication. The rapid development of innovative information and communications technology (ICT) has enabled and enhanced the capacity for large-scale data collection, analysis and dissemination. As exemplified during COVID-19, such systems have allowed individuals to remotely conduct normative daily tasks and maintain social cohesion despite extreme physical distancing measures [38]. ICT allows sectors to continue their basic functions, such as the health sector using telemedicine for non-essential patients, the education sector using remote learning, and the business sector to promote teleworking. Furthermore, technology has enabled sectors to conduct extraordinary functions in the context of a pandemic beyond national jurisdiction. For example, governments and private entities have implemented efficient surveillance, reporting, or contact tracing through artificial intelligence other technologies that aggregate and share large-scale data; mapping disease spread for community protection [24]. However, in adopting ICT measures, careful considerations must be made to ensure digital tools are inclusive to all members of the community. For example, barriers of access and adaptability must be considered within ICT infrastructure to guarantee access to information and services among the elderly, disabled groups, lower-income households, or those living in remote areas [6].

### 3.6. Knowledge Product Marketing

Updating and generating new recommendations and tools for DRR is a continuous process. Outside of science, these tools can be used to develop effective public communication strategies and raising awareness for community preparedness [6]. The DRR community requires more tools and knowledge-sharing platforms to facilitate planning and strategy development [2], and there is as yet limited availability of updated and relevant DRR knowledge product specialization for biological hazards at a global scale such as the COVID-19 hazards. This has hindered knowledge sharing, scenario planning, and cross-sectoral learning. Although the WHO Thematic Platform for Health-EDRM was formed in September 2016 to “coordinate activities, promote information-sharing, develop partnerships, and provide technical advice to strengthen the Health-EDRM research field”, as of 2020, there remains an urgent need to strengthen multidisciplinary learning and collaborative efforts to maximize the impact of such knowledge development. Active engagement in shared knowledge and building understanding of the complex nature of biological hazards will enable the DRR community to develop and facilitate scientific risk assessment mechanisms so as to build resilient systems in the future [3].

## 4. Discussion

The COVID-19 pandemic has had devastating human and socioeconomic impacts worldwide. The global attention received for COVID-19 provides an opportunity for the health and DRR communities to reconceptualize knowledge and tools for disaster risk mitigation, response and recovery. In November 2020, DRR experts from the Asia-Pacific region attended the Asia-Pacific Partnership for Disaster Risk Reduction (APP-DRR) Forum to share progress, policy priorities and opportunities thus far for DRR in the region with respect to the COVID-19 pandemic [11]. Experts shared learnings for risk governance, including health system resilience; data management; residual risk management, risk communication, digital literacy, and knowledge product marketing. Special academic attention has been paid regarding the integration of biological hazards into DRR planning [6]. Although the Health-EDRM Framework was established to ensure that health will be considered within the DRR dialog at intra, inter and multidisciplinary levels, further efforts are required to ensure that both health and non-health actors in education and continuous education are included within DRR frameworks. Notably, and urgently, to include students and young professionals who will become key stakeholders in the next decade in promoting awareness, scientific development, policy, and capacity at the intersection of health and DRR. Successful implementation of the Sendai Framework will require updated Health-EDRM and DRR tools that consider concurrent, cascading and interacting hazards. Cascading risks have a serious impact on national action plans, and the impacts faced are becoming increasingly complex and interdependent. However, national plans still tend to focus on the most probable impacts rather than on those that will bring the most complex consequences that require heavily coherent and coordinated response [7]. Adaptive governance mechanisms are necessary for building interdependent resilience cutting across social, institutional, economic and ecological levels. Reinforcing continuous learning and innovation across the governance of different sectors will strengthen DRR outcomes [39], systematic risks analysis and related action planning. Table 1 summarizes how the above discussion may expand into and impact DRR, related challenges and suggested solutions.

The COVID-19 pandemic has demonstrated the ability for a biological hazard to travel across national borders and the need for governance structures mitigating against transnational risks. There is a role for North-South, and South-South collaboration in jointly developing technological, medical and social innovations, which can accommodate local variation, that lead to creating incentivization for long-term multi-generational resilience [40]. Inter-sectoral coordination such as public–private partnership models for health service provision should be explored to maximize the functionality of service provision and the range of services available [6].

There is a large number of activities, priorities and stakeholders that must be mobilized, facilitated, and coordinated, not only in response to the pandemic and in the recovery phase but also in developing DRR plans against the next hazard that emphasizes a coordinated response across linked sectors rather than over-burdening one sector [6]. In order to operationalize lessons learned in impactful, cost-effective and sustainable ways, methods in cross-program planning, monitoring and evaluation can be taken from the area of project management. This will involve viewing international development as a transformative public sector project when evaluating delivery constraints such as time, cost and quality. International development and private sector projects are at risk of facing similar challenges in poor stakeholder management, cost overruns, inadequate monitoring, and lack of understanding of local context. However, international development projects often have less tangible goals and certainly face higher socio-political complexities that induce further transaction costs [41].

## 5. Conclusions

COVID-19 has impacted progress across the Sustainable Development Goals (SDG). The economic impact has resulted in an estimated 71 million people pushed into extreme poverty (SDG 1—no poverty); 80 million children under the age of 1 are estimated to miss routine vaccinations (SDG 3—good health and wellbeing); school closures will affect 90% of students (SDG 4—quality education); cases of domestic violence will increase in 30% of countries (SDG 5—gender equality); and 60% of countries will experience prison overcrowding and further risk of spreading COVID-19 (SDG 16—peace, justice and strong institutions) [40].

However, the pandemic also creates opportunities to strengthen SDG, such as strengthening partnerships under SDG 17 in developing shared warning mechanisms, data sharing, multi-stakeholder partnerships in science to build evidence-based policy recommendations. These partnerships can be built between sectors of a country, but also in North–South or South–South cooperation [6].

Opportunities and resources available during the response and recovery of the COVID-19 pandemic may allow DRR stakeholders to examine and evaluate systemic weaknesses in a holistic and comprehensive manner. To ensure that the global population would be more sufficiently protected against future concurrent, cascading, or even interacting hazards, revisiting current DRR plans and strategies within the current framework of biological hazards will be instrumental. The COVID-19 pandemic has created a chance to strengthen partnerships; build mechanisms for a coordinated response between DRR experts and counterparts in health; and build health as a core component across disaster prevention, mitigation, response and recovery. Building understanding of the multifaceted and adaptive components of risk governance within people in their formative years will allow the next-generation to accelerate towards achieving the targets under the Sendai Framework as well as the SDGs. Continuous education, notably of students and young professionals, may be a key component when building awareness about DRR.

## Figures and Tables

**Table 1:** Opportunities for different areas to expand into and impact disaster risk reduction (DRR), related challenges and suggested solutions.

Issues	Opportunities to Expand into DRR	Challenges	Suggested Solution
Health systems resilience	• Strengthen health considerations within multi-sectoral national or international DRR action plans	• Weaknesses in current action plans that do not consider the entire disaster cycle or prepare for concurrent, cascading or interacting risks	• Develop multi-hazard, multi-sectoral and adaptive action plans for DRR
	• Improve hazard-related health outcomes by revaluating the resilience and vigilance of the health system as a whole	• Weaknesses in current action plans that do not consider multi-sectoral impact or response • Weaknesses in current action plans that do not consider post-epidemic long-term physiological or psychological effects	• Consider health systems-wide paradigm to care, beyond clinical care
		• National bodies are creating unique, siloed national action plans that lack complementarity	• Reinforce awareness-building and continuing professional education as a key component in policy development
Data management	• Identify areas of improvement in existing data platforms (collection, storage, analysis, sharing) in terms of: - Inclusivity of vulnerable groups - Compatibility with other DRR information platforms - Compatibility with technological advancement	• Security considerations in terms of data storage and management	• Consider inclusivity and representation of vulnerable groups in building data management tools
		• Ethical considerations for data use, monetization of data, and personal data protection	• Incorporate the latest technological advancement and adaptive capacities for piloting secure data collection and data management tools
	• Unique opportunity to collect robust post-pandemic data across all populations, to be used in recovery assessment research or for future hazards		• Continuous education regarding systems development and updates
Residual risk management	• Define or redefine “essential” groups, including part-time workers, nonprofessionals (e.g., home care givers), and non-health sector workers	• There is no standard definition of “essential” workers or nonprofessional workers	• Develop policy and guidelines to protect essential workers and nonprofessional workers
	• Research into health impact and health needs of a pandemic on essential workers and nonprofessional workers, in order to build evidence-based policy and guidelines	• Lacking recognition or political will to protect the health and wellbeing of these groups (e.g., material provision, information dissemination)	• Data and research in health impact and needs of essential workers and nonprofessional groups including needs in material resources, information gaps, or training opportunities
			• Continuous education of stakeholders involved in policy update and development
Risk communication	• Review or strengthen top-down government approaches to early warning systems	• Limited evidence of barriers to inclusivity of populations or inclusivity of communication channels	• Develop inclusive platforms for information dissemination (e.g., used by the elderly, disabled individuals)
	• Consider health literacy in disaster risk communication and decision-making frameworks	• Limited but growing political will in managing misinformation or in determining the reliability of the information	• Community dialog to review and research barriers of information access and understanding
	• Consider demographic and health factors (e.g., old age, physical disabilities) in ability access to information		• Building awareness and appropriate policies for communities facing vulnerabilities and improving patterns of communication under complex circumstances
Digital literacy	• Use of novel technology to develop tools for DRR data management (e.g., information sharing, data collection, tracking)	• Complex access to digital tools for certain groups (e.g., elderly, remote/rural groups, low-income groups)	• Build community dialog to promote the use of digital tools and understand barriers to usage
	• Use novel technology to improve health DRR (e.g., diagnostics, telemedicine)		• Pilot novel and innovative tools for telemedicine, robotic temperature monitoring, or automated dispensary
			• Building awareness and appropriate policies for communities facing vulnerabilities and improving patterns of communication under complex circumstances
Knowledge product marketing	• Update Health-EDRM and DRR tools, in particular, to consider the multifaceted and adaptive nature of concurrent, cascading and interacting hazards	• Lack of political or institutional will for multi-sectoral planning	• Collect evidence and lessons learned for needs in addressing novel biological hazards
	• Multi-sectoral participation in the development of updated tools and guidelines		• Develop adaptive tools and knowledge products
			• Begin a multi-sectoral dialog for DRR
			• Building awareness and identifying knowledge gaps within communities to encourage active research and policy development
